# Turnover of Grassland Roots in Mountain Ecosystems Revealed by Their Radiocarbon Signature: Role of Temperature and Management

**DOI:** 10.1371/journal.pone.0119184

**Published:** 2015-03-03

**Authors:** Jens Leifeld, Stefanie Meyer, Karen Budge, Maria Teresa Sebastia, Michael Zimmermann, Juerg Fuhrer

**Affiliations:** 1 Agroscope, Climate/Air Pollution Group, Zurich, Switzerland; 2 Institute for Geography, Friedrich Schiller Universität, Jena, Germany; 3 Independent Researcher, Kirkwall, Orkney, United Kingdom; 4 Forest Sciences Centre of Catalonia, Lleida, Spain; 5 Dept. HBJ, ETSEA, University of Lleida, Lleida, Spain; 6 University of Natural Resources and Life Sciences Vienna, Department of Forest and Soil Sciences, Vienna, Austria; University of Western Sydney, AUSTRALIA

## Abstract

Root turnover is an important carbon flux component in grassland ecosystems because it replenishes substantial parts of carbon lost from soil via heterotrophic respiration and leaching. Among the various methods to estimate root turnover, the root’s radiocarbon signature has rarely been applied to grassland soils previously, although the value of this approach is known from studies in forest soils. In this paper, we utilize the root’s radiocarbon signatures, at 25 plots, in mountain grasslands of the montane to alpine zone of Europe. We place the results in context of a global data base on root turnover and discuss driving factors. Root turnover rates were similar to those of a subsample of the global data, comprising a similar temperature range, but measured with different approaches, indicating that the radiocarbon method gives reliable, plausible and comparable results. Root turnover rates (0.06–1.0 y^-1^) scaled significantly and exponentially with mean annual temperatures. Root turnover rates indicated no trend with soil depth. The temperature sensitivity was significantly higher in mountain grassland, compared to the global data set, suggesting additional factors influencing root turnover. Information on management intensity from the 25 plots reveals that root turnover may be accelerated under intensive and moderate management compared to low intensity or semi-natural conditions. Because management intensity, in the studied ecosystems, co-varied with temperature, estimates on root turnover, based on mean annual temperature alone, may be biased. A greater recognition of management as a driver for root dynamics is warranted when effects of climatic change on belowground carbon dynamics are studied in mountain grasslands.

## Introduction

Root turnover denotes the annual replenishment of roots that die, are grazed or sloughed off by physical forces, and replaced with newly developed roots. The input of root material to soil, together with exudation from living roots, is a key component in the soil-plant carbon (C) cycle, as 20–30 percent of the net primary production (NPP) in temperate grassland ecosystems is allocated to the standing root biomass [1]. Furthermore, organic matter from root turnover is an important factor for the stabilization of C in soil because root litter is, on average, significantly more resistant to microbial decomposition than aboveground litter [2, 3]. Therefore, reliable estimates of rates and drivers of C input into soil by roots are essential for the understanding of the terrestrial C flux.

Root turnover can be studied by multiple approaches [4, 5]. From these methods, the radiocarbon approach has not been widely applied, mainly because of high analytical costs. The radiocarbon approach utilizes the natural atmospheric radiocarbon (^14^C) signal, which has been altered by humans with the spiking of the atmosphere with so-called bomb ^14^C. For studying roots, the atmospheric ^14^C signal, which is incorporated into the plant through photosynthesis and transferred to roots, is particularly valuable as it provides a ubiquitous tracer of C dynamics for the relevant time span of years to decades [6]. In comparison to physical methods, isotopic approaches allow the study of root turnover by means of the roots’ isotopic signature after excavation, without prior disturbance of the system (e.g. as with ingrowth cores and minirhizotrons) or the need for comparison of different subunits of the system as in the case of sequential soil and root excavation.

Previous work has indicated that root turnover is sensitive to site mean annual temperature (MAT) [7]. Their global analysis of root turnover from different biomes, including many grassland sites, revealed that MAT was the only significant predictor for root turnover, although the primary mechanisms may be related to radiation flux and length of the growing season rather than temperature itself [8]. Because of the close relationship between root turnover and temperature, the latter is used to estimate root turnover as part of the grassland C budget both in experimental studies [9] and vegetation modeling [10, 11, 12].

However, it has been shown that root turnover, in grasslands, is also responsive to management intensity via fertilization, cutting, grazing, or prescribed burning [13, 14, 15, 16]. If management practices, such as harvest frequency or stocking density, are related to temperature, an estimation of root turnover based on temperature alone may be biased. A co-regulation of management for root turnover, in addition to temperature, may be particularly notable for mountain grasslands at and above the montane zone, where topographic and climatic constraints reduce agricultural options and lead to less intensive management. For example, along altitudinal gradients in the Swiss Alps, cutting frequencies of hay meadows typically decline from 2–4 cuts per year below 1000 m asl, to only one cut above 2000 m asl [17], with corresponding changes in above ground productivity and nutrient exports. Permanent meadows and pastures cover 3360 Mha or ca. 68% of the global agricultural area and are often natural or semi-natural, hence non-intensively managed. In Europe (excluding Russian Federation), permanent meadows and pastures account for a smaller share (c. 34% or 85 Mha) of the agricultural area [18]. These ecosystems are often man-made, maintained via cutting and grazing, and, in productive lowlands, often intensively utilized. In contrast, mountain grasslands in humid Central and Western and drier Southern mountain ranges, together accounting for c. 42% of the European grassland area, are less intensively managed [19].

Further examination and clarification of how temperature and management intensity drive root turnover alone or in interaction would be valuable for projections on the effect of climate change on root and soil carbon dynamics. Over- or underestimation of root turnover may have critical consequences for our understanding of processes guiding grassland soil C sequestration. However, root turnover in mountain ecosystems has only been measured in a few case studies and, furthermore, how these turnover rates compare to the results from other grassland biomes is unknown.

Here we address the following hypotheses:

- Radiocarbon based methods provide reliable root turnover estimates.
- Mean annual temperature is an important factor in controlling root turnover in mountain grasslands.
- Root turnover rates are influenced by management intensity.

We addressed the above hypotheses by using new and partially published data from our research on root turnover from various grassland ecosystems in different mountain regions of Europe. Furthermore, we put our data into a global context by comparing our results to that of a previously published global data set on root turnover [7].

## Sites and Methods

Soil samples were taken with 4–8 replicates each from 25 mountain grasslands in the Alps and the Pyrenees (Table 1). Most sampling plots are below the timber line and all plots represent decadal or centennial grassland use. Three plots (“Wallis 4”, “Furka”, “Pyrenees 4”) are situated at or above the tree line and hence represent alpine tundra. For most plots steel cores of 7 cm diameter with plastic liner were used for sampling. For plots in the Pyrenees a rectangular auger with 4 cm edge widths and 20 cm length was used. After excavation, samples were cut into sections of 0–5, 5–10, 10–20, and 20–30 cm. At each site field replicates were taken situated at horizontal distances of 5–15 m to each other. The plots span a range of MATs of 0.0 to 10.6°C, with different management types and intensities as well as varying soil characteristics (Table 1). Details on plots “Wallis” can be found in Leifeld et al. [20], on plot “Furka” in Budge et al. [21], on plots “Pyrenees” in Budge [22], on plot “Alp Flix” in Leifeld et al. [23], and on plots “Stubai” and “Vinschgau” in Meyer et al. [24] and Meyer [25]. Root radiocarbon data from plots “Wallis”, “Pyrenees”, and two plots from “Stubai” have not been published before. Plot management details were made available by scientist who have been working on these plots for longer periods. In many mountain grasslands combined management by grazing and haying is common. Based on the available information we classified the management intensities, using indicators for the magnitude of biomass removal, into high (2–4 cuts per year or stocking density of > 2 livestock units (LU) and grazing for most of the growing season), medium (1–2 cuts per year or stocking density of 1–2 LU with grazing for not more than 2 months of the growing season), and low (max. 1 cut per year, occasional grazing with < 1 LU or unmanaged with wild life grazing only. The latter type is representative of alpine tundra). The Livestock unit (LU) is a unit used to compare or aggregate numbers of animals of different species or categories per hectare. Equivalences based on the food requirements of the animals are defined. By definition, a cow weighing 600 kg and producing 3000 litres of milk per year = 1 LU [26]. This study did not involve endangered or protected species and it was not necessary to obtain specific permission to sample at any of the sampling plots.

**Table 1:** Plot characteristics (C coordinates, E elevation [m asl], MAT mean annual temperature [°C]; MAP mean annual precipitation [mm], soil clay content [%], soil pH, soil C/N ratio, MT management type [G grazing, M cut meadow, GM mixed use, U unmanaged], MI management intensity [**H** high 2–4 cuts per year, high stocking density with > 2 LU, **M** medium 1–2 cuts per year, low stocking density with1–2 LU, **L** max. 1 cut per year, occasional grazing or unmanaged with wild life grazing only; i.e. max. 1 LU], pMC percent modern carbon, MRT root mean residence time, year of sampling).

	C	E	MAT	MAP	clay	pH	C/N	MT	MI	pMC	MRT	year	n
Wallis 1	46.25°N 7.92°E	990	7.8	658	21	6.8	10.1	GM	H	106.5	2.0	2005	6
Wallis 2		1410	5.7	857	20	6.6	10.4	GM	M	107.7	4.0	2005	6
Wallis 3		1795	3.9	1039	23	5.4	10.7	GM	M	108.0	4.5	2005	6
Wallis 4		2200	0.9	1231	24	3.8	12.2	U	L	111.0	8.4	2005	8
Furka	46.56°N 8.40°E	2564	0.0	1890	16	3.9	16.3	U	L	108.6	15.5	2007	6
Pyrenees 1	42.25°N 1.80°E	853	10.6	906	45	7.4	10.5	G	M	102.2	1.0	2008	8
Pyrenees 2		1279	8.6	865	37	7.3	11.3	G	M	106.4	4.2	2008	8
Pyrenees 3	42.25°N 1.68°E	1817	5.8	917	52	7.1	12.4	G	M	108.7	7.7	2008	8
Pyrenees 4		2293	3.9	816	44	6.0	11.9	G	L	109.3	8.5	2008	8
Alp Flix 1	46.52°N 9.67°E	2100	2.2	1050	29	3.9	13.0	G	L	108.0	7.5	2009	4
Alp Flix 2		2100	2.2	1050	29	4.7	15.3	G	L	109.1	9.0	2009	4
Alp Flix 3		2100	2.2	1050	33	4.7	13.1	G	L	108.6	8.4	2009	4
Alp Flix 4		2100	2.2	1050	26	4.8	14.2	G	L	113.5	16.7	2009	4
Alp Flix 5		2100	2.2	1050	31	5.3	11.7	G	L	106.9	5.9	2009	4
Alp Flix 6		2100	2.2	1050	31	5.5	12.0	G	L	107.8	7.1	2009	4
Alp Flix 7		2100	2.2	1050	34	5.7	11.7	G	L	107.6	6.9	2009	4
Alp Flix 8		2100	2.2	1050	31	5.9	12.1	G	L	107.4	6.6	2009	4
Stubai 1	47.13°N 11.30°E	1070	6.5	825	18	6.3	9.2	M	H	104.8	1.0	2009	6
Stubai 2		1380	5.1	933	17	4.6	10.7	M	M	106.1	3.6	2009	6
Stubai 3		1850	3.0	1097	13	4.9	10.0	GM	M	105.9	3.3	2009	6
Stubai 4		1950	3.0	1097	21	5.5	10.0	G	M	105.3	2.0	2009	6
Stubai 5		2000	3.0	1097	23	5.4	12.0	U	L	106.1	4.3	2009	6
VG 1^1^	46.71°N 10.64°E	1860	6.6	527	17	4.9	12.7	G	H	106.1	1.0	2009	4
VG 2		1890	6.6	527	21	5.8	11.1	M	M	104.7	3.6	2009	4
VG 3		1790	6.6	527	15	5.0	11.0	U	L	106.0	4.0	2009	4

Last column indicates number of field sample replicates. Field sample replicates were pooled for ^14^C analysis. Soil values refer to 0–10 m soil depth.

^1^ VG = Vinschgau

After soil sample excavation roots were carefully separated by washing and rinsing the sample followed by picking roots with a pair of tweezers. Roots were washed thereafter in nylon bags with 63 micron mesh, dried at 40°C and weighed. Roots were not further separated by size but mostly consisted of material with less than 1 mm in diameter. We aimed at isolating only living roots on which we decided by visual inspection based on root color and integrity. The C and N content of roots and corresponding soil samples was determined on finely ground samples by elemental analysis (Hekatech Euro CHN, Germany). Soil pH was measured with an electrode in an 1:2.5 soil:0.01 M CaCl_2_ water suspension and soil clay content with the pipette method after H_2_O_2_ treatment and dispersion of the soil-water suspension with Na-polyphosphate. Root radiocarbon content was determined at the AMS facility of Laboratory of Ion Beam Physics of the Institute for Particle Physics of at the ETH (the Swiss Federal Institute of Technology), Zurich [27]. The results were expressed as percent modern carbon (pMC) calculated following the protocol of Stuiver and Polach [28]. We used composite root samples from the field replicates per plot and studied the root ^14^C signature for 0–10 cm for all plots and at deeper layers for five of the plots. Samples were taken between 2005 and 2009 (Table 1) and radiocarbon activities were corrected for radioactive decay between sampling and measurement year (1–2 years).

### Estimating root carbon turnover from radiocarbon data

Two approaches for estimating root C turnover, hereafter referred to as root turnover, were applied following Gaudinski et al. [29]. Both make use of the change in atmospheric ^14^C content, as a result of nuclear bomb testing, mainly in the 1960s.

**Firstly**, we used the time dependent steady-state radiocarbon turnover model developed by Harkness et al. [30], on which later steady-state models were built, including the first application to estimate the mean residence time of fine roots [29]. In brief, the model assumes a continuous exchange of ^14^C between the respective C pool (here root) and the atmosphere. The temporal change in radiocarbon signature of the C pool depends on the atmospheric signature and the mean residence time of root C:

$$A_{t}=A_{\left(t-1\right)}e^{-k}+(1-e^{-k})A_{\left(i\right)}-A_{\left(t-1\right)}\lambda$$ [1]

where A_(t)_ is the (measured) ^14^C activity (pMC) of C in roots at time t, A_(t-1)_ the ^14^C activity of the previous year; A(_i_) is the atmospheric ^14^C activity, *k* the exchange rate constant of root C, and λ the ^14^C decay constant (1/8267 y; [28]). With this model we do not consider any time lag between C assimilation and fixation in the root biomass. Values for A_i_ were taken from the atmospheric ^14^C record of Stuiver et al. [31] for the period from 1511 to 1954 and from Levin and Kromer [32] for the period 1959 to 2003. The period between 1954 and 1959 was linearly interpolated. Data for 2004–2010 were taken from Levin et al. [33]. The model gives *k* (the turnover rate) or its inverse 1/*k* (the mean residence time). For one plot, repeatedly taken root samples were available and used to test the reliability of the model assumptions (S1 Fig.).

In the **second** approach, the corresponding point, in the atmospheric ^14^C curve, that equals the root’s radiocarbon signature is determined (hereafter called curve reading, leading to root age). Unlike the steady state model, the curve reading is based on the assumption that the C in the standing root biomass is fixed only at the time span of root formation and no further C exchange with the atmosphere (via assimilates) occurs. Our comparison of the curve reading, representing a highly skewed age distribution, and the steady state model, representing an age distribution with overlying first order decay dynamics and continuous exchange, gives a sense of the bias inherent to the choice of method (see e.g. [4]).

For both approaches the accuracy of the AMS measurement must be set in context to the annual change in atmospheric ^14^C. Our samples, taken during the first decade of this century, were measured with an average two-sigma uncertainty of 0.40 pMC compared to a mean annual atmospheric decline of 0.48 pMC. We therefore consider calculated mean residence times of below one year as below the detection limit and set these values to one. Thus root mean residence times of one year may be an overestimation of the true mean residence time, under the conditions of the applied model.

### Statistics

The relationship between root turnover rate and MAT was evaluated using non-linear regression. To account for management effects, a general linear model (GLM) was computed using site as main factor, management intensity as factor nested within site, mean annual temperature (MAT) as random effect and root turnover rate as response variable. Detailed results of the GLM model can be found in the supplementary material (S1 Table).

## Results and Discussion

### Method choice

**Fig 1:**
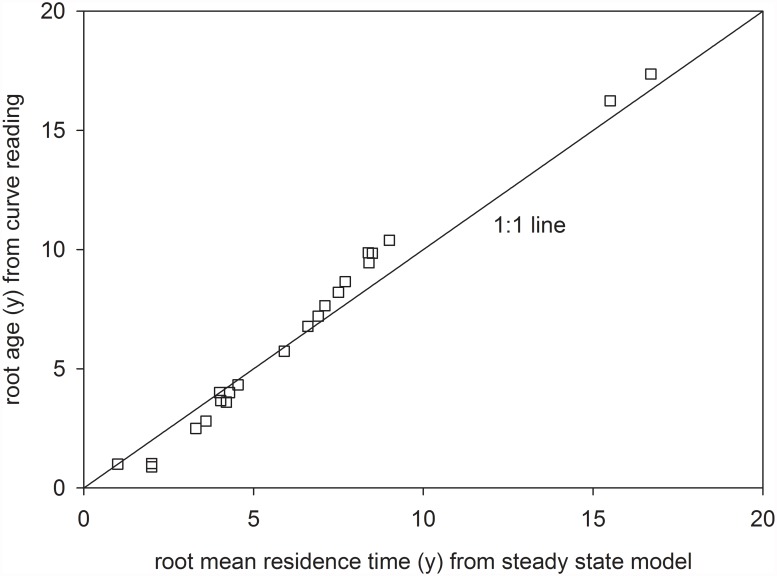
Comparison of root mean residence times estimated from steady-state turnover modeling (x axis) and root age as estimated from curve reading of the atmospheric radiocarbon bomb peak (y axis) for depth 0–10 cm.

The two approaches for estimating root C mean residence times revealed similar results that were highly significantly correlated (r = 0.99, P<0.001; Pearson’s) and did not deviate systematically from the 1:1 line (Fig. 1). Differences between the two methods were not significant (paired t-test), indicating that model choice has a minor influence on estimating root mean residence time although in single cases differences may be pronounced. Short-lived roots tend to have longer calculated mean residence times, when the steady-state model is used, whereas long lived roots tend to have longer mean residence times with the curve reading. This discrepancy is related to the shape of the atmospheric bomb peak, with its declining radiocarbon activity during recent decades: Longer lived roots still possess a higher, less recent radiocarbon activity that, in the case of the curve reading, will be attributed to a higher root age. For practical purposes, the choice of the model seems of minor importance. In the following we refer to the results of the steady-state model.

### Temperature effects

**Fig 2:**
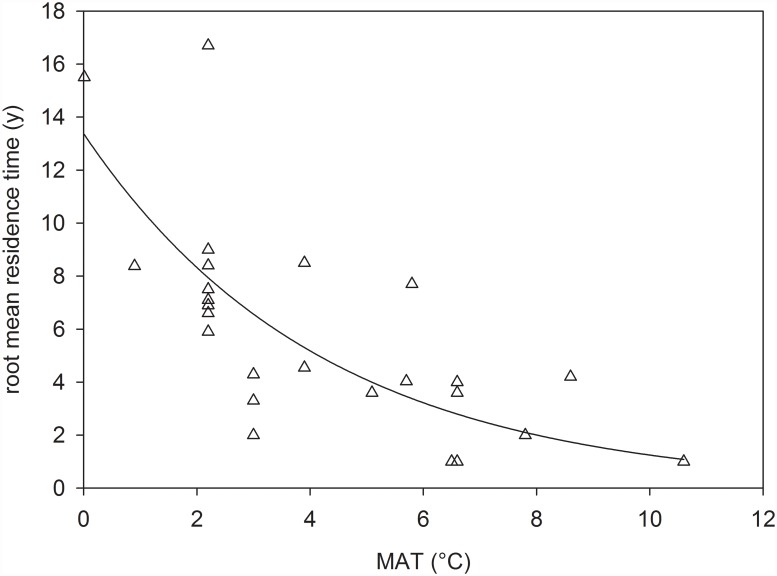
Mean annual temperature and root carbon mean residence time (0–10 cm) of all sampling plots. The curve displays the exponential relationship mean residence time (y) = 13.38 [1.94] * exp(-0.24 [0.05] * x), R2 = 0.53,P < 0.001. Values in square brackets are 1 SE.

Our data indicate a range of root C mean residence times (0–10 cm soil depth) of between 1 to 17 years for a gradient in MAT of 0 to 11°C (Fig. 2). The highly significant exponential relationship between root mean residence time and MAT suggest that temperature exerts important control on root turnover. Root mean residence times were converted to root turnover rate (1/MRT) to make them comparable to the global data set published by Gill and Jackson [7]. Our data indicate an apparent temperature dependency of root turnover of Q10 = 7.2 whereas, on the contrary, Gill and Jackson [7] report a Q10 of only 1.6 for their global grassland data that encompass all climatic zones (Table 2). When only the sites in Gill and Jackson [7], whose MAT lie within the range of our data set are considered (i.e. reduced data set, temperature range -0.7–12.0°C), their corresponding Q10 becomes 1.9, which is still much below that of our measurements. MATs’ did not differ significantly between our results and the reduced data set in Gill and Jackson [7] (t-test).

**Table 2:** Overview of apparent temperature sensitivities for root turnover rates (y^-1^)in i) the current data set, ii) the global data set for grassland encompassing all climatic zones (Gill and Jackson [7]) and iii) a reduced data set extracted from Gill and Jackson [7] for grassland spanning a range in MAT of -0.7–12°C which corresponds to the range in MAT in the current data set. Exponents are given (± 1 SE).

	Current data set	Gill & Jackson global data set	Gill & Jackson reduced data set^1^
exponent	0.197 (0.045)	0.048 (0.007)	0.063 (0.041)
apparent Q10	7.20	1.62	1.88

^1^ no significant temperature effect

Our results, from mountain grasslands, reveal turnover rates of between 0.06 and 1.0 y^-1^. These are similar to the range for grasslands comprising the reduced data set derived from Gill and Jackson [7]. The studies compiled in [7] were mostly based on total root biomass production, using sequential soil coring, as well as on measurements with minirhizotrones and ingrowth cores. The similarity to results from our measurements suggests that the estimation of root turnover rates in grasslands, of the selected MAT range, is not biased towards the selected methodology. Our data are also in line with results from temperate grasslands by Solly et al. [14], applying the same ^14^C approach, who reported mean root ages (‘root age’ in [14] is synonymous to the term ‘root mean residence time’ as used in this study) of 0.7–2.6 years for a MAT range of 6.0–8.5°C.

### Management effects

The temperature sensitivity for root turnover, derived by Gill and Jackson [7], is based on a comprehensive, well examined data set, comprising a wide range of environmental conditions. However, grassland management intensities were not provided for their data set. We argue that the seemingly high temperature sensitivity in our data may be related to other drivers that co-vary with temperature. In mountain ecosystems, temperature typically decreases with elevation (in our case, elevation explained 77% of the variability in MAT with a decrease in MAT by 0.53°C per 100 m altitude). Grasslands at higher elevations are managed less intensively because of poor accessibility and locally steeper slopes. Data in Table 1 confirm systematic differences in management intensity along the elevation gradient. Plots with high to medium management intensity were, on average, located at 1512 m asl with an MAT of 6.1°C and a mean root turnover rate of 0.48 y^-1^, whereas plots with low management intensity were on average situated at 2127 m asl, MAT 2.5°C, mean root turnover rate 0.14 y^-1^. This difference in root turnover between high to medium management intensity on the one hand and low management intensity on the other, is higher as would be expected with a Q10 of 1.9, as derived from the reduced data set in Gill and Jackson [7]. For a MAT of 6.1°C, the reduced data set would induce a root turnover rate of only 0.18 y^-1^ relative to a rate of 0.14 y^-1^ at 2.5°C. Statistical results from the GLM indicate that management intensity is an important factor controlling root turnover in the studied grasslands (S1 Table, S2 Fig.). While temperature alone explained 53% of the variability (Fig. 2), consideration of site (i.e, plots grouped by region, see supplement) and three management intensities substantially improved the prediction of root turnover rates (R2 = 0.70). The analysis further indicated, that high and medium, but not low intensity management tends to accelerate root turnover, suggesting that temperature as a single predictor variable is particularly useful for natural and semi-natural grasslands, but may reveal biased estimates for managed grasslands.

Management intensity comprises two elements, the magnitude of biomass removal as indicated by cutting frequency or stocking density, and the fertilization level. How does **biomass removal** regulate turnover of grassland roots? Using ^13^C pulse labelling technique Crawford et al. [16] reported that grass cutting resulted in lower aboveground NPP compared to an uncut control, but in a higher allocation of assimilated C to roots. For simulated grazing vs. non grazing in pasture soils from France, Klumpp et al. [34] observed significantly different root mean residence times (0.9 years under high grazing vs. 2.3 years without grazing using continuous labeling). A stimulating effect of grazing on root turnover compared to an ungrazed exclosure was also reported for a temperate grassland in Argentina [35]. However, Garcia-Pausas et al. [36] found high variability in total annual root production between grazed and ungrazed (grazing exclosure) plots, indicating that grazing effects on root turnover are site specific. For two subalpine land use gradients within our study plots, Meyer et al. [24] found evidence for an intensity dependent root turnover using natural ^14^C. Roots under intensive pasture or meadow revealed shorter mean residence times than those of abandoned grassland or less intensively managed pasture. Stimulation of root turnover by higher stocking densities or cutting frequencies as indicated by our data from mountain grasslands seems to represent a general picture and is mostly in line with findings from other grassland ecosystems.

Beyond its direct effect on root productivity and turnover via biomass removal, management intensity exerts control on the **soil’s nutrient status**, which may have further repercussions on soil biological activity. An indicator for the soil nutrient content in our data set is soil nitrogen and the corresponding soil C/N ratio (Table 1). A wider C/N ratio of soil organic matter is indicative for a larger fraction of non decomposed plant litter, corresponding to a low N status and low microbial activity. Root mean residence times (0–10 cm) correlate highly significantly (r = 0.85, P < 0.001) with the soil’s C/N ratio. This suggests in soils with wide C/N ratio and thus low biological activity, roots turn over at lower rates. An indirect effect of nutrient status on root turnover was also discussed by Solly et al. [14] for grasslands under different fertilization practices in Germany. In their study mean root ages were significantly greater at sites with lower fertilization levels and higher plant diversity. The authors reasoned that with higher nutrient addition, an accelerated root turnover is mediated through or accompanied by the establishment of a different vegetation community. A positive effect of nutrient addition on root turnover was also reported for a New Zealand pasture soil [15].

**Fig 3:**
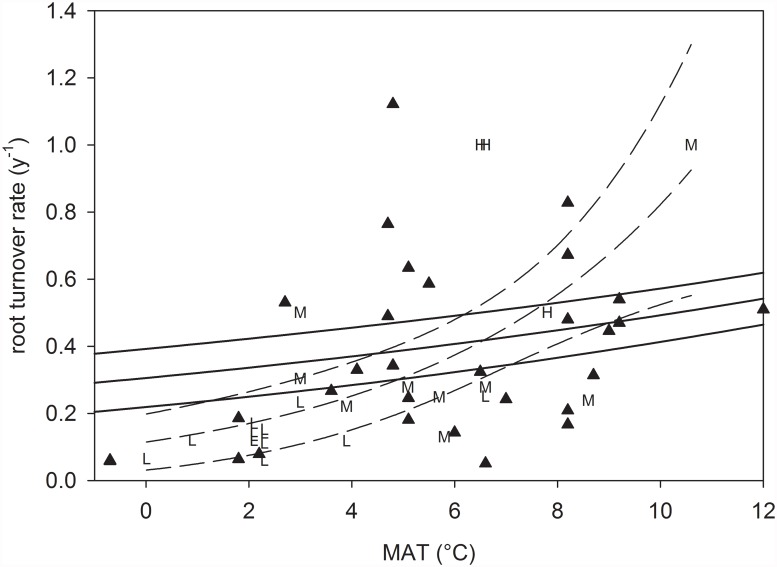
Comparison of root turnover rates (y^-1^) between the current data set (dashed lines) and the global data set from grassland soils in Gill and Jackson [7] (for explanation, please see text; triangles and solid lines). Data from the current data set are displayed as letters which refer to the management intensity of the plots (see Table 1). Only the temperature range relevant for this study is shown. Envelopes are 95% confidence intervals of the regression lines.

Together, management intensity may stimulate root turnover in grasslands via biomass removal and/or fertilization. Our results indicate that, for the same MAT, turnover tends to be faster with more intensive management (Fig. 3) and hence would support this hypothesis. However, because of the non-controlled nature of our study, this interpretation must be taken with caution.

### Root turnover at different depths

**Fig 4:**
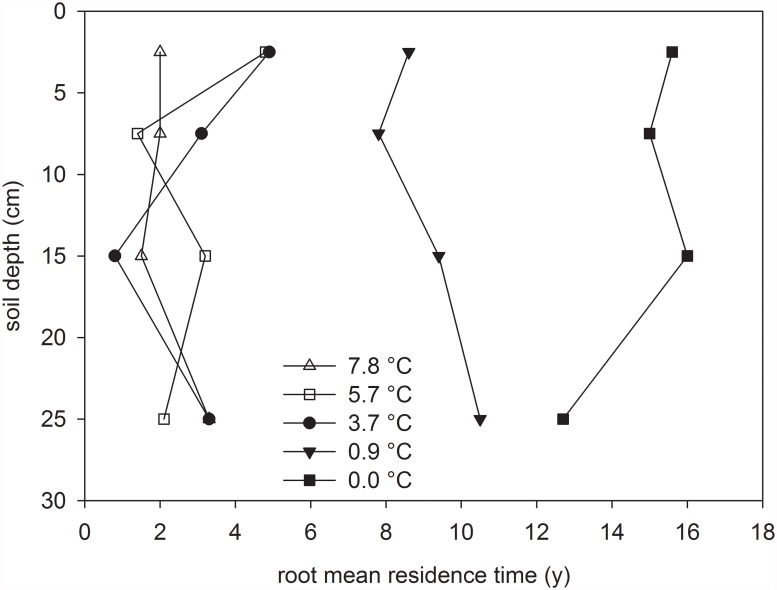
Depth distribution of root mean residence times along a gradient in mean annual temperature from + 7.8°C to 0.0°C (corresponding to plots “Wallis” and “Furka” in Table 1. Lines are a guide for the eyes only.

To study whether mean residence times derived from topsoil measurements are valid also for deeper roots, we made use of ^14^C measurements from four consecutive depth intervals available for five plots (Fig. 4). Root mean residence times showed no distinct depth related trend at any of the plots albeit some variability was observed. Normalization to the upper layer values allowed to pool data from these five plots and regress them against depth. There was no significant depth trend in root mean residence time for these five plots (r = 0.01, P = 0.97). Similar to our results but for plants rooting down to > 5 m, Trumbore et al. [6] found no evidence for an increase in root age with depth in tropical forest and pasture soils. On the other hand, Gaul et al. [37] reported a significant increase in fine root age in a Norway Spruce stand with depth (0–0.5 m), suggesting that depth-age relationships for roots may depend on vegetation type. Our measurements in mountain grasslands indicate that radiocarbon dating of surface layer roots can also be used to calculate C inputs from root turnover in deeper soil without inducing a systematic error.

## Conclusions

Bomb radiocarbon was used to estimate root mean residence times in grasslands from the Alps and the Pyrenees. The corresponding turnover rates are in the range of a previously published global database and therefore, the method seems reliable and the results are plausible. The acute temperature sensitivity of root turnover observed in our data set, as compared to the global numbers, is also related to management intensity. With more intensive management, occurring mostly in warmer regions of mountain ecosystems, root turnover rates increase more than would be expected from temperature alone. In contrast, low root turnover rates in non-intensively managed regions at the subalpine-alpine transition are mainly regulated by temperature. Our results do not preclude factors other than temperature or management in the influence of root turnover in these ecosystems but there is evidence that they both exert important control. Hence, predictions of future soil carbon stocks, using models where root turnover is parameterized solely with temperature, may produce biased estimates for those grassland ecosystems where management intensity scales with MAT.

## Supporting Information

S1 Fig Graphical visualization of the applied steady-state turnover model for calculating root mean residence times. The line displays the best solution to equation (1) in the paper, and symbols represent repeated measurements of roots from plot Stubai 1 in 2002, 2005, and 2008 (root mean residence time 1.2 y). Error bars are the AMS 2σ uncertainty.
(TIF)

S2 Fig Comparison of root turnover rates (ln k) measured by the ^14^C turnover model (for details, see paper), and two statistical models. Open triangles: Prediction based on MAT alone (equivalent to Fig. 2), crosses: Prediction based on the GLM according to S1 Table.
(TIF)

S1 Table Results of the general linear model with site as main factor, management intensity as factor nested within site and mean annual temperature (MAT) as random effect for root turnover rates in mountain grasslands. (DOCX)

## References

[1] Bolinder MA, Janzen HH, Gregorich EG, Angers DA, Vandenbygaart AJ. An approach for estimating net primary productivity and annual carbon inputs to soil for common agricultural crops in Canada. Agric Ecosyst Environ. 2007;118: 29–42.

[2] Crow SE, Lajtha K, Filley TR, Swanston CW, Bowden RD, Caldwell BA. Sources of plant-derived carbon and stability of organic matter in soil: implications for global change. Glob Chang Biol. 2009;15: 2003–2019.

[3] Rasse DP, Rumpel C, Dignac MF. Is soil carbon mostly root carbon? Mechanisms for a specific stabilisation. Plant Soil 2005;269: 341–356.

[4] Luo YQ. Uncertainties in interpretation of isotope signals for estimation of fine root longevity: theoretical considerations. Glob Chang Biol. 2003;9: 1118–1129.

[5] Milchunas D. Estimating root production: Comparison of 11 methods in Shortgrass Steppe and review of biases. Ecosystems 2009;12: 1381–1402.

[6] Trumbore S, Da Costa ES, Nepstad DC, De Camargo PB, Martinelli L, Ray D. et al Dynamics of fine root carbon in Amazonian tropical ecosystems and the contribution of roots to soil respiration. Glob Chang Biol. 2006; 12: 217–229.

[7] Gill RA, Jackson RB. Global patterns of root turnover for terrestrial ecosystems. New Phytol. 2000; 147: 13–31.

[8] Fitter AH, Graves JD, Self GK, Brown TK, Bogie DS, Taylor K. Root production, turnover and respiration under two grassland types along an altitudinal gradient: influence of temperature and solar radiation. Oecologia 1998;114: 20–30. 28307553 10.1007/s004420050415

[9] Kucharik CJ, Fayram NJ, Cahill KN. A paired study of prairie carbon stocks, fluxes, and phenology: comparing the world’s oldest prairie restoration with an adjacent remnant. Glob Chang Biol. 2006; 12: 122–139.

[10] Riedo M, Grub A, Rosset M, Fuhrer J. A pasture simulation model for dry matter production, and fluxes of carbon, nitrogen, water and energy. Ecol Modell. 1998;105: 141–183.

[11] Gill RA, Kelly RH, Parton WJ, Day KA, Jackson RB, Morgan JA. et al Using simple environmental variables to estimate belowground productivity in grasslands. Glob Ecol Biogeogr Lett. 2002;11:79–86.

[12] Soussana J-F, Maire V, Gross N, Bachelet B, Pages L, Martin R. et al Gemini: A grassland model simulating the role of plant traits for community dynamics and ecosystem functioning. Parameterization and evaluation. Ecol Modell. 2012;231: 134–145.

[13] von Haden AC, Dornbush ME. Patterns of root decomposition in response to soil moisture best explain high soil organic carbon heterogeneity within a mesic, restored prairie. Agric Ecosyst Environ. 2014;185: 188–196.

[14] Solly E, Schoening I, Boch S, Mueller J, Socher SA, Trumbore SE et al Mean age of carbon in fine roots from temperate forests and grasslands with different management. Biogeosciences 2013;10: 4833–4843.

[15] Scott JT, Stewart DPC, Metherell AK. Alteration of pasture root carbon turnover in response to superphosphate and irrigation at Winchmore New Zealand. N Z J Agric Res. 2012;55: 147–159.

[16] Crawford MC, Grace PR, Oades JM. Effect of defoliation of medic pastures on below-ground carbon allocation and root production In: Lal R, Kimble J, Follett RF, Stewart BA, editors. Management of carbon sequestration in soil, 1997; Boca Raton CRC Press pp. 381–389.

[17] Dietl W. Pflanzenbestand, Bewirtschaftungsintensität und Ertragspotential von Dauerwiesen. Schweizerische Landwirtschaftliche Monatshefte 1986;6: 241–261.

[18] FAOSTAT. Food and Agriculture Organization of the United Nations, available: http://faostat3.fao.org/ Accessed 2014 September 24.

[19] Pfimlin A, Todorov NA. Trends in European forage systems for milk and meat production: facts and new concerns. Grassland Science in Europe 2003;8: 1–10.

[20] Leifeld J, Zimmermann M, Fuhrer J, Conen F. Storage and turnover of carbon in grassland soils along an elevation gradient in the Swiss Alps. Glob Chang Biol. 2009;15: 668–679.

[21] Budge K, Leifeld J, Hiltbrunner E, Fuhrer J. Alpine grassland soils contain large proportion of labile carbon but indicate long turnover times. Biogeosciences 2011;8: 1911–1923.

[22] Budge K. Does carbon distribution and turnover in (sub)alpine grassland soils indicate these areas may be potential carbon dioxide hotspots in the event of global warming? PhD Dissertation University of Zurich, 2011. Available: http://opac.nebis.ch/ediss/20121419.pdf.

[23] Leifeld J, Bassin S, Conen F, Hajdas I, Egli M, Fuhrer J. Control of soil pH on turnover of belowground organic matter in subalpine grassland. Biogeochemistry 2013;112: 59–69.

[24] Meyer S, Leifeld J, Bahn M, Fuhrer J. Free and protected soil organic carbon dynamics respond differently to abandonment of mountain grassland. Biogeosciences 2012;9: 853–865.

[25] Meyer S. Land-use changes in subalpine grasslands: Effects of abandonment on soil organic carbon storage and dynamics. PhD dissertation University of Zurich, 2012. Available: http://opac.nebis.ch/ediss/20131614.pdf.

[26] European Commission. Agriculture and Environment. Available: http://ec.europa.eu/agriculture/envir/report/en/lex_en/report_en.htm Accessed 2015 January 8.

[27] Synal H-A, Stocker M, Suter M. MICADAS: A new compact radiocarbon AMS system. Nucl Instrum Methods Phys Res B 2007;259: 7–13.

[28] Stuiver M, Polach HA. Reporting of C-14 data—discussion. Radiocarbon 1977;19: 355–363.

[29] Gaudinski JB, Trumbore SE, Davidson EA, Cook AC, Markewitz D, Richter DD. The Age of Fine-Root Carbon in Three Forests of the Eastern United States Measured by Radiocarbon. Oecologia 2001;129: 420–429. 28547197 10.1007/s004420100746

[30] Harkness DD, Harrison AF, Bacon PJ. The temporal distribution of bomb C-14 in a forest soil. Radiocarbon 1986;28: 328–337.

[31] Stuiver M, Reimer PJ, Braziunas TF. High-precision radiocarbon age calibration for terrestrial and marine samples. Radiocarbon 1998;40: 1127–1151.

[32] Levin I, Kromer B. The Tropospheric (CO_2_)-C-14 Level in Mid-Latitudes of the Northern Hemisphere (1959–2003). Radiocarbon 2004;46: 1261–1272.

[33] Levin I, Kromer B, Hammer S. Atmospheric Delta (CO2)-C-14 trend in Western European background air from 2000 to 2012. Tellus B Chem Phys Meteorol. 2013;65.

[34] Klumpp K, Fontaine S, Attard E, Le Roux X, Gleixner G, Soussana J-F. Grazing triggers soil carbon loss by altering plant roots and their control on soil microbial community. J Ecol. 2009;97: 876–885.

[35] Pucheta E, Bonamici I, Cabido M, Diaz S. Below-ground biomass and productivity of a grazed site and a neighbouring ungrazed exclosure in a grassland in central Argentina. Austral Ecology 2004;29: 201–208.

[36] Garcia-Pausas J, Casals P, Romanyà J, Vallecillo S, Sebastià MT. Seasonal patterns of belowground biomass and productivity of grazed and ungrazed mountain grasslands in the Pyrenees. Plant Soil 2011;340: 315–326.

[37] Gaul D, Hertel D, Leuschner C. Estimating fine root longevity in a temperate Norway spruce forest using three independent methods. Funct Plant Biol. 2009; 36: 11–19. 10.1071/FP08195 32688623

