# Supplementary figures and images for: Turnover of Grassland Roots in Mountain Ecosystems Revealed by Their Radiocarbon Signature: Role of Temperature and Management
Source: PLoS One. 2015 Mar 3;10(3):e0119184. doi: 10.1371/journal.pone.0119184 (PMC4347979; doi:10.1371/journal.pone.0119184)

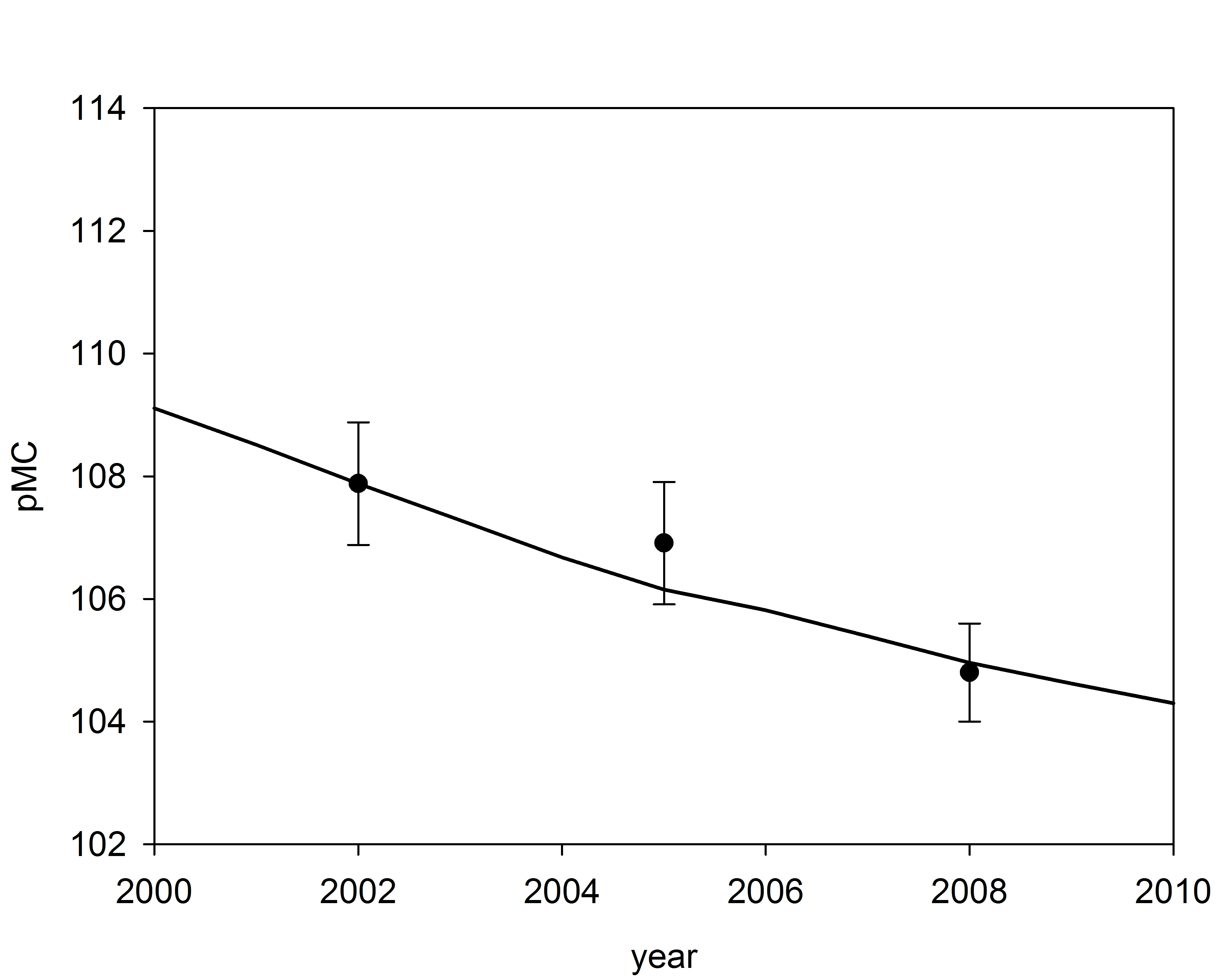

Supplement: S1 Fig — The line displays the best solution to equation (1) in the paper, and symbols represent repeated measurements of roots from plot Stubai 1 in 2002, 2005, and 2008 (root mean residence time 1.2 y). Error bars are the AMS 2σ uncertainty. (TIF) [file pone.0119184.s001.TIF]

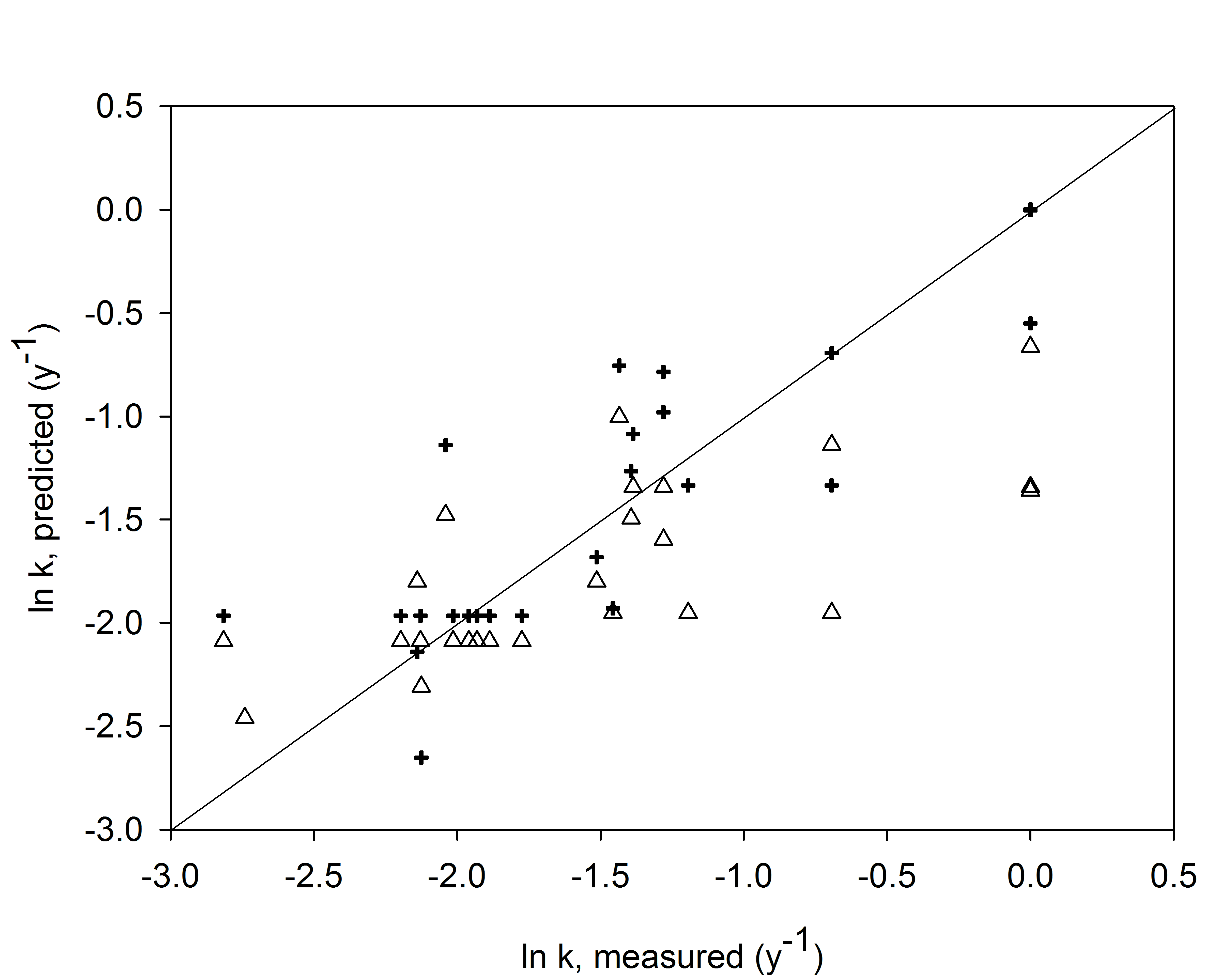

Supplement: S2 Fig — Open triangles: Prediction based on MAT alone (equivalent to Fig. 2), crosses: Prediction based on the GLM according to S1 Table. (TIF) [file pone.0119184.s002.TIF]
