# Supplementary material for: Turnover of Grassland Roots in Mountain Ecosystems Revealed by Their Radiocarbon Signature: Role of Temperature and Management
Source: PLoS One. 2015 Mar 3;10(3):e0119184. doi: 10.1371/journal.pone.0119184 (PMC4347979; doi:10.1371/journal.pone.0119184)
Supplement: S1 Table — (DOCX) [file pone.0119184.s003.docx]

Table S1. Results of the general linear model with site as main factor, management intensity as factor nested within site and mean annual temperature (MAT) as random effect for root turnover rates in mountain grasslands. All sampling plots were grouped using their geographical coordinates as variables to derive three clusters considered as factor ‘site’ in the GLM. The general equation is

*k* = ‘I’+ ‘level name’ + MAT*’MAT’

where acronyms in quotation marks refer to parameter values. Letters h, m, and l stand for different management intensities (see text), and letters a-c for three geographical clusters, i.e., sites (see below). Empty cells (-) denote factor combinations not occurring in the field.

|  |  |  |  | | | Model parameters | | | | | | |  | | |
| --- | --- | --- | --- | --- | --- | --- | --- | --- | --- | --- | --- | --- | --- | --- | --- |
|  | | | | |  | | level name | |  | | *k* parameter value | |  |  |  |
|  | | | | |  | |  | |  | |  | |  |  |  |
| Intercept | | | | |  | | ‚I‘ | |  | | -0.0154 | |  |  |  |
| a low | | | | |  | | ‚al‘ | |  | | 0.0377 | |  |  |  |
| a medium | | | | |  | | ‚am‘ | |  | | -0.0073 | |  |  |  |
| a high | | | | |  | | ‘ah’ | |  | | 0.0970 | |  |  |  |
| b low | | | | |  | | ‚bl‘ | |  | | -0.0767 | |  |  |  |
| b medium | | | | |  | | ‚bm‘ | |  | | 0.0254 | |  |  |  |
| b high | | | | |  | | - | |  | | - | |  |  |  |
| c low | | | | |  | | - | |  | | - | |  |  |  |
| c medium | | | | |  | | ‚cm‘ | |  | | 0.1183 | |  |  |  |
| c high | | | | |  | | ‚ch‘ | |  | | 0.6648 | |  |  |  |
| MAT | | | | |  | | ‚MAT‘ | |  | | 0.0535 | |  |  |  |
|  | | | | |  | |  | |  | |  | |  |  |  |
| Model performance | | | | | | | | | | | | | | | |
| Multiple R2 | | | | Adjusted R2 | | | SS model | df model | | SS residual | | df residual | | F | p |
| 0.80 | | | | 0.70 | | | 1.565 | 8 | | 0.398 | | 16 | | 7.857 | 0.000261 |
|  | | | |  | | |  |  | |  | |  | |  |  |
| Univariate significance | | | | | | | | | | | | | | | |
|  | | | |  | | | SS | df | |  | |  | | F | p |
| Intercept | | | |  | | | 0.0074 | 1 | |  | |  | | 0.2965 | 0.594 |
| Intensity | | | |  | | | 0.5011 | 5 | |  | |  | | 4.0252 | 0.015 |
| Site | | | |  | | | 0.2378 | 2 | |  | |  | | 4.7744 | 0.024 |
| MAT | | | |  | | | 0.098 | 1 | |  | |  | | 3.9383 | 0.064 |
| Error | | | |  | | | 0.025 | 16 | |  | |  | |  |  |

The assignment of sampling plots from Table 1 in the main text to one of three site clusters a-c applying k-means clustering is:

Site in field geographical cluster

Wallis 1 a

Wallis 2 a

Wallis 3 a

Wallis 4 a

Furka a

Pyrenees 1 b

Pyrenees 2 b

Pyrenees 3 b

Pyrenees 4 b

Alp Flix 1 a

Alp Flix 2 a

Alp Flix 3 a

Alp Flix 4 a

Alp Flix 5 a

Alp Flix 6 a

Alp Flix 7 a

Alp Flix 8 a

Stubai 1 c

Stubai 2 c

Stubai 3 c

Stubai 4 c

Stubai 5 c

VG 1 c

VG 2 c

VG 3 c
